# Hospital and community wastewater as a source of multidrug-resistant ESBL-producing *Escherichia coli*

**DOI:** 10.3389/fcimb.2023.1184081

**Published:** 2023-05-15

**Authors:** Lenka Davidova-Gerzova, Jarmila Lausova, Iva Sukkar, Kristina Nesporova, Lucie Nechutna, Katerina Vlkova, Katerina Chudejova, Marcela Krutova, Jana Palkovicova, Jakub Kaspar, Monika Dolejska

**Affiliations:** ^1^ Central European Institute of Technology, University of Veterinary Sciences Brno, Brno, Czechia; ^2^ Department of Biology and Wildlife Diseases, Faculty of Veterinary Hygiene and Ecology, University of Veterinary Sciences Brno, Brno, Czechia; ^3^ Department of Microbiology, Faculty of Medicine and University Hospital Pilsen, Charles University, Pilsen, Czechia; ^4^ Biomedical Center, Faculty of Medicine, Charles University, Pilsen, Czechia; ^5^ Department of Medical Microbiology, 2nd Faculty of Medicine, Charles University and Motol University Hospital, Prague, Czechia; ^6^ Center of Cardiovascular and Transplant Surgery, St. Anne’s University Hospital Brno, Brno, Czechia; ^7^ Department of Clinical Microbiology and Immunology, Institute of Laboratory Medicine, The University Hospital Brno, Brno, Czechia

**Keywords:** Escherichia coli, antibiotic resistance, wastewater, beta-lactamases, whole-genome sequencing

## Abstract

**Introduction:**

Hospitals and wastewater are recognized hot spots for the selection and dissemination of antibiotic-resistant bacteria to the environment, but the total participation of hospitals in the spread of nosocomial pathogens to municipal wastewater treatment plants (WWTPs) and adjacent rivers had not previously been revealed.

**Methods:**

We used a combination of culturing and whole-genome sequencing to explore the transmission routes of *Escherichia coli* from hospitalized patients suffering from urinary tract infections (UTI) *via* wastewater to the environment. Samples were collected in two periods in three locations (A, B, and C) and cultured on selective antibiotic-enhanced plates.

**Results:**

In total, 408 *E. coli* isolates were obtained from patients with UTI (n=81), raw hospital sewage (n=73), WWTPs inflow (n=96)/outflow (n=106), and river upstream (n=21)/downstream (n=31) of WWTPs. The majority of the isolates produced extended-spectrum beta-lactamase (ESBL), mainly CTX-M-15, and showed multidrug resistance (MDR) profiles. Seven carbapenemase-producing isolates with GES-5 or OXA-244 were obtained in two locations from wastewater and river samples. Isolates were assigned to 74 different sequence types (ST), with the predominance of ST131 (n=80) found in all sources including rivers. Extraintestinal pathogenic lineages frequently found in hospital sewage (ST10, ST38, and ST69) were also found in river water. Despite generally high genetic diversity, phylogenetic analysis of ST10, ST295, and ST744 showed highly related isolates (SNP 0-18) from different sources, providing the evidence for the transmission of resistant strains through WWTPs to surface waters.

**Discussion:**

Results of this study suggest that 1) UTI share a minor participation in hospitals wastewaters; 2) a high diversity of STs and phylogenetic groups in municipal wastewaters derive from the urban influence rather than hospitals; and 3) pathogenic lineages and bacteria with emerging resistance genotypes associated with hospitals spread into surface waters. Our study highlights the contribution of hospital and municipal wastewater to the transmission of ESBL- and carbapenemase-producing *E. coli* with MDR profiles to the environment.

## Introduction

Hospital and urban wastewater gather sewage enriched with a huge proportion of antibiotic-resistant bacteria (ARB) and transfer those to municipal wastewater treatment plants (WWTPs) (Servais & Passerat, 2009). Treated wastewater represents epidemiologically important hotspots where selective pressure on both resistant and sensitive populations intervene in favor of resistant bacteria, due to the presence of antimicrobial residue (Amos et al., 2014; Buelow et al., 2018; Manaia et al., 2018). During the treatment processes in WWTPs, bacteria are largely eliminated, yet a proportion of viable cells including ARB infiltrate adjacent surface waters and spread to the environment (Karkman et al., 2018). In rivers, ARB and antibiotic resistance genes (ARGs) are mixed with freshwater bacteria and from there could be introduced to wildlife living in/near water sources (Dolejska and Literak, 2019), mediating their further spread to the environment. Dissemination of antibiotic resistance poses a great risk for further disease treatment and must be epidemiologically monitored.

*Escherichia coli* is a mammalian fecal commensal bacterium that also includes pathogenic variants. Extraintestinal pathogenic *E. coli* (ExPEC) strains are known as leading uropathogens. They have frequent resistance to clinically important antimicrobials including cephalosporins and carbapenems, and they are also very often multi-drug resistant (Manges et al., 2019a). More than 14,300 different *Escherichia*/*Shigella* sequence types (STs) have been described so far (1.3.2023; Zhou et al., 2020) but only a small proportion of particular ExPEC lineages, such as ST10, ST38, ST69, ST131, etc., are globally connected to nosocomial infections and life-threatening bacteremia (Manges et al., 2019a). However, the precise mechanism of ExPECs dissemination remains unclear (Riley, 2014).

The aim of this study was to determine whether antimicrobial-resistant *E. coli* from hospitalized patients suffering from urinary tract infections (UTI) and affiliated hospital wastewater share similar characteristics with strains isolated from municipal WWTPs and adjacent rivers. We mainly wanted to explore 1) resistance phenotypes, ARGs, plasmids, and virulence-associated genes (VAGs) in collected *E. coli* strains; 2) distribution of the most prevalent sequence types (STs) including ExPEC strains across sample types; and 3) the link between different ecological niches *via* the examination of phylogenetic analysis of *E. coli*. For this purpose, cultivation and whole-genome sequencing (WGS) of pre-selected resistant *E. coli* were combined to evaluate their spread to adjacent rivers *via* single nucleotide polymorphism (SNP) analysis.

## Materials and methods

### Sample collection

Sampling was performed in three large cities (A, B, and C) across the Czech Republic in August 2020 (at locations A and B) and April 2021 (at locations A, B, and C) to cover possible variations and seasonality. Anonymized clinical isolates of cephalosporin-resistant *E. coli* were collected from hospitalized patients (other than those hospitalized in infection diseases departments) for 2 weeks during the routine diagnostics of UTI. Environmental samples consisted of 1) hospital wastewater (composed of outflow of hospital WWTPs where only wastewater from infection diseases departments are treated and raw hospital sewage from the rest of the hospital) from three cities (A, B, and C); 2) municipal wastewaters from two cities (A and B) at the inflow and outflow of WWTPs before it enters surface waters; and 3) river water collected at the WWTPs upstream and downstream (A and B). Just before the first sampling at location A, local floods occurred and the river was not accessible, so we had to perform sampling at the first possible access point to the river water which was up to 1,9 km downstream. At location C, inflow and outflow of municipal WWTPs as well as river water was missing due to disagreement on sampling conditions between the WWTP facility and our institution.

All water samples were collected in sterile one-liter glass flasks on the last day of collection of clinical isolates. Wastewater sampling was performed on Tuesdays at approximately 9 o’clock to cover the average wastewater population and to exclude the early morning peak or weekend flushes. Samples were transported to the laboratory, kept at 4 °C, and processed immediately.

### Cultivation of resistant *E. coli* and antibiotic susceptibility testing

Clinical isolates were inoculated on MacConkey agar (MCA; Oxoid, UK) with cefotaxime (2 mg/L) or meropenem (0.125 mg/L) to obtain ESBL- and carbapenemase-producing strains. Species identification was confirmed using MALDI-TOF mass spectrometer (Microflex LT, Bruker Daltonics, Germany) and purified isolates were subsequently stored at -80 °C.

Water samples were first serially ten-fold diluted or concentrated using filtering (0.22 μm; Sigma-Aldrich, US) before plating (depending on presumed amount of viable bacterial colonies in the various types of samples) and then cultivated in triplicate on chromogenic medium Brilliance™ *E. coli*/coliform Selective Agar (Oxoid, UK) at 37 °C overnight. Suspected *E. coli* isolates were subcultured on MCA with cefotaxime or meropenem to obtain ESBL- and carbapenemase-producing isolates. Up to 30 resistant *E. coli* colonies per water sample were collected and identified using the MALDI-TOF mass spectrometer and stored at -80 °C.

Isolates selected on media with meropenem during the first sampling period and identified as *E. coli* were all subjected to WGS. However, only 1/101 isolates contained carbapenemase-encoding genes. Therefore, isolates obtained from the second sampling were processed with a modified approach in order to decrease the sequencing cost. After the selection on meropenem-supplemented media they were tested for carbapenemase-encoding genes by PCR (**Supplement Table 1**) and only isolates presumptively containing the gene (9/156) were used for further analysis including WGS.

Beta-lactamase production was tested in all *E. coli* isolates by Mastdiscs combi Carba plus D73C (Mast Diagnostics, UK). A minimal inhibitory concentration (MIC) to the 24 antimicrobials (MIC G-I, MIC G-II) was determined (Erba Lachema, Czech Republic). Isolates were categorized as resistant, intermediate, or susceptible to each antimicrobial according to EUCAST (if available) and for chloramphenicol, tetracycline, cefoperazone, and netilmicin CLSI breakpoints were used (**Supplement Table 2**). Isolates showing resistance to at least one antimicrobial agent within three or more groups of ATBs were considered MDR (Magiorakos et al., 2012).

### Whole-genome sequencing

DNA of all selected *E. coli* isolates (n=408) was extracted using NucleoSpin Tissue kit (Macherey-Nagel, Germany) and subjected to library preparation and sequencing (HiSeq 4000, Illumina, Inc., USA) following the manufacturer’s instructions. Data was processed as described previously (Kutilova et al., 2021). Raw Illumina paired-end reads were adapted and quality (Q≥20) trimmed by Trimmomatic v0.39 (Bolger et al., 2014) and high-quality trimmed reads were assembled using the *de novo* assembler SPAdes v3.13.1 (Bankevich et al., 2012). Assemblies were deposited in the GenBank (Bioproject no. PRJNA938932).

### Genomic data analysis

Obtained assemblies were analyzed by ABRicate v0.9.8 (Seemann T, Abricate; available at https://github.com/tseemann/abricate) using database ResFinder (2022-05-24) (Bortolaia et al., 2020) and specific chromosomal mutations using python-based PointFinder (2022-08-08) (Zankari et al., 2017) to identify the most common ARGs. Results of antimicrobial susceptibility testing were compared to the expected antimicrobial susceptibility based on WGS data. The level of their concordance was evaluated as 1) “gene absence” for the situation when the phenotypic resistance was observed for specific antibiotics but no relevant genetic marker was detected in WGS data or 2) “gene presence” when a genetic marker expected to provide the resistance to specific antibiotic according to the ResFinder database was detected but the observed phenotype was susceptible. Chromosomal point mutations whose phenotype have not been assigned yet (“unknown mutations”) were examined when no expected genetic markers for beta-lactams or quinolones were detected. For beta-lactams except carbapenems, any kind of beta-lactamase encoding gene or chromosomal mutations in *ampC* was evaluated as sufficient explanation for the observed resistance. In the case of chloramphenicol, we detected *catB3* gene with lower coverage than was our threshold coverage (90%) but we considered this partial gene presence as sufficient explanation for observed resistance to these antibiotics.

Database PlasmidFinder (2022-03-30) (Clausen et al., 2018) using ABRicate was used to analyze the presence of plasmid replicons while pMLST analysis was performed to determine plasmid replicon sequence type (RST). ColV plasmid presence was identified based on previously reported criteria (Liu et al., 2018) which considers a strain to be ColV-positive if it carries at least one or more genes from four or more of the following six gene sets: *cvaABC* and *cvi* (the ColV operon), *iroBCDEN* (the salmochelin operon), *iucABCD* and *iutA* (the aerobactin operon), *etsABC*, *ompT* and *hlyF*, and *sitABCD*.

ABRicate using Virulence factor database (core dataset, version 2022-12-23) (Liu et al., 2019) and VirulenceFinder 2.0 (Tetzschner et al., 2020) were used to assign the isolates as ExPECs. Such isolates were defined as those harboring two or more of the six following VAGs: *papA* and/or *papC* (P fimbriae), *sfa*/*focDE* (S and F1C fimbriae), *afa*/*draBC* (Dr-binding adhesins), *kpsM* II (group 2 capsule), and *iutA* (aerobactin receptor) (Peirano et al., 2013). ST131 clades were distinguished based on allelic variants of *fimH, parC, gyrA*, and *bla*_CTX-M_ (Petty et al., 2014; Pitout & Finn, 2020). ST131 virotypes were determined based on the presence or absence of *afa*/*draBC*, *iroN*, *sat*, *ibeA*, *papGII/III*, *cnf1*, *hlyA*, *ctdB*, *neuC-K1*, and *kpsM*K2/K5 into virulence types (Dahbi et al., 2013). Additionally, we examined 43 selected VAGs previously described by Kim et al. (2021) to be associated with UTI. These genes (operons/gene groups) covered adhesion factors (*ecp*, *hcp*, *eaeH*, *fim*, *pap*, *foc*, *afa*, *sfa*, *cfa*, *fae*, *aap/aspU*, *eae*, and *paa*), autotransporter systems (*upaG*/*ehaG*, *ehaB*, *agn43*, *tsh*, *sat*, *vat*, *cah*, *eae*X, *cdi*, *upaH*, *pic*, *ehaA*, *pet*, *aatA*, and *espl*), invasion proteins (*ibeABC* and*tia*), indirect iron uptake systems (*sitA*, *chuA*, *fyuA*, *iucC*, ireA, and *iroN*), and toxins (*hlyE*, *usp*, *senB*, *cnf1*, *hlyABD*, *cdtB*, and *astA*). ARGs, plasmid replicons, and VAGs were considered present if length coverage and identity to the reference sequence were ≥90%. Sequence types and phylogenetic groups (PGs) of E. coli isolates were determined by MLST 2.0 (Larsen et al., 2012; Seemann T, mlst; available at https://github.com/tseemann/mlst) and ClermonTyping tools (Beghain et al., 2018), respectively. Serotype was determined using SerotypeFinder 2.0 (Joensen et al., 2015) with O:H typing database and fimbriae type was defined by *fimH* typing (Roer et al., 2017).

### Phylogenetic and SNP analysis

From the assemblies, a collection of 408 prokka-annotated sequences was generated and analyzed with PIRATE 1.0.4 to infer core and pangenomes of our *E. coli* collection. A maximum-likelihood tree was built using RAxML and the phylogenetic tree was further visualized in iTOL (Letunic & Bork, 2021, version 6.6). Afterwards, the matrix of SNP distances was identified by running snp-dists 0.6.3 (available at Seemann, T., snp-dists; https://github.com/tseemann/snp-dists) on the core gene alignment, generating a core SNP alignment. The distance between two *E. coli* isolates from different sources lower than ≤10 SNP threshold was evaluated as clonal spread (Schürch et al., 2018).

Individual phylogenetic trees were constructed for predominating lineages ST131, ST10, ST38, ST69, and ST1927. Moreover, we enhanced our dataset of ST1972 with five isolates from the previous study (2016; Bioproject PRJNA943108, unpublished data) and the above described data-analysis was performed to compare with data from this study. Information about the number of core genome genes is provided in **Supplement Table 3** together with overall matrix and separate matrices for selected STs.

### Statistical analysis

Basic data analysis and visualization were performed in Excel (Microsoft). Comparison of categorical variables of VAGs prevalence using Fisher exact test was conducted in R Core Team (2022, version 2022.07.2 + 576) as well as further data computing and visualization. A p-value <0.05 was considered statistically significant.

## Results

### Antibiotic resistance phenotypes of *E. coli* isolates

**Figure 1:**
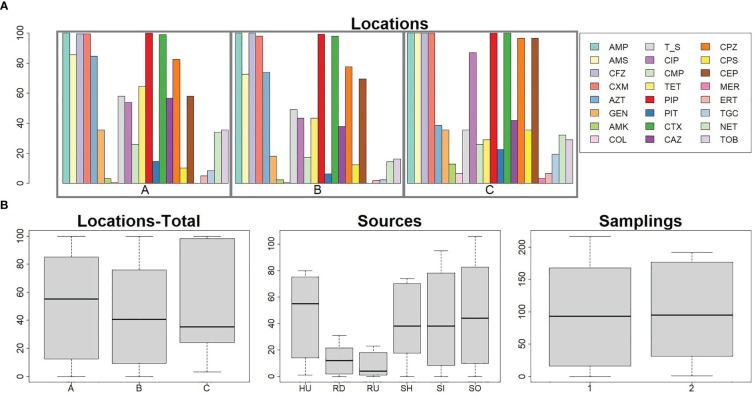
Numbers of antibiotic-resistant (*E*) *coli* isolates across locations, sources, and sampling periods. Panel **(A)**: Percentage resistances of *E. coli* isolates against 24 antibiotics across three locations A, B, and C Bar chart, see legend for different antibiotics prevalence. Panel **(B)**: Prevalence of antibiotic resistance based on location without consideration of different sources or particular antibiotics (location A n=216, location B n=160, location C n=32), source (HU n=81, SH n=73, SI n=96, SO n=106, RU n=21, RD n=31), and sampling period (sampling period 1 n=217, sampling period n=191). Box and whiskers plots show median and quartiles in antibiotic resistance based on different characteristics. Sources HU – UTI isolates, RD – downstream river water, RU – upstream river water, SH – hospital wastewater, SI -WWTP inflow, SO – WWTP outflow. Number of isolates in Location A: UTI (n=42), SH (n=30), SI (n=55), SO (n=56), RU (n=12), RD (n=21). Location B: UTI (n=30), SH (n=20), SI (n=41), SO (n=50), RU (n=9), RD (n=10). Location C: UTI (n=9), SH (n=23). Antibiotic AMK, amikacin; AMP, ampicillin; AMS, ampicillin + sulbactam; AZT, aztreonam; CAZ, ceftazidime; CEP, cefepime; CFZ, cefazolin; CIP, ciprofloxacin; CMP, chloramphenicol; COL, colistin; CPS, cefoperazone + sulbactam; CPZ, cefoperazone; CTX, cefotaxime; CXM, cefuroxime; ERT, ertapenem; GEN, gentamicin; MER, meropenem; NET, netilmicin; PIP, piperacillin; PIT, piperacillin + tazobactam; T/S, co-trimoxazole; TET, tetracycline; TGC, tigecycline; TOB, tobramycin.

A collection of 408 *E. coli* samples was obtained from clinical (n=81), raw hospital sewage (n=73), WWTPs inflow (n=96) and outflow (n=106), and river upstream (n=21, only the first sampling period) and downstream (n=31) of WWTPs. Most isolates produced ESBL enzymes (96%) and showed an MDR profile (96%) with resistance ranging from five to 23 antibiotics. Apart from beta-lactams, isolates were resistant to tetracyclines (222/408, 54%), trimethoprim/sulfamethoxazole (214/408, 52%), ciprofloxacin (175/408, 43%), and other antibiotics (**Figure 1A**). The percentage of obtained resistant colonies from one sample differed between locations and sources but was consistent during the sampling periods (**Figure 1B**; **Supplement Table 2**).

### Antibiotic resistance genes of whole-genome sequenced *E. coli* and correlation to phenotypic susceptibility testing

**Figure 2:**
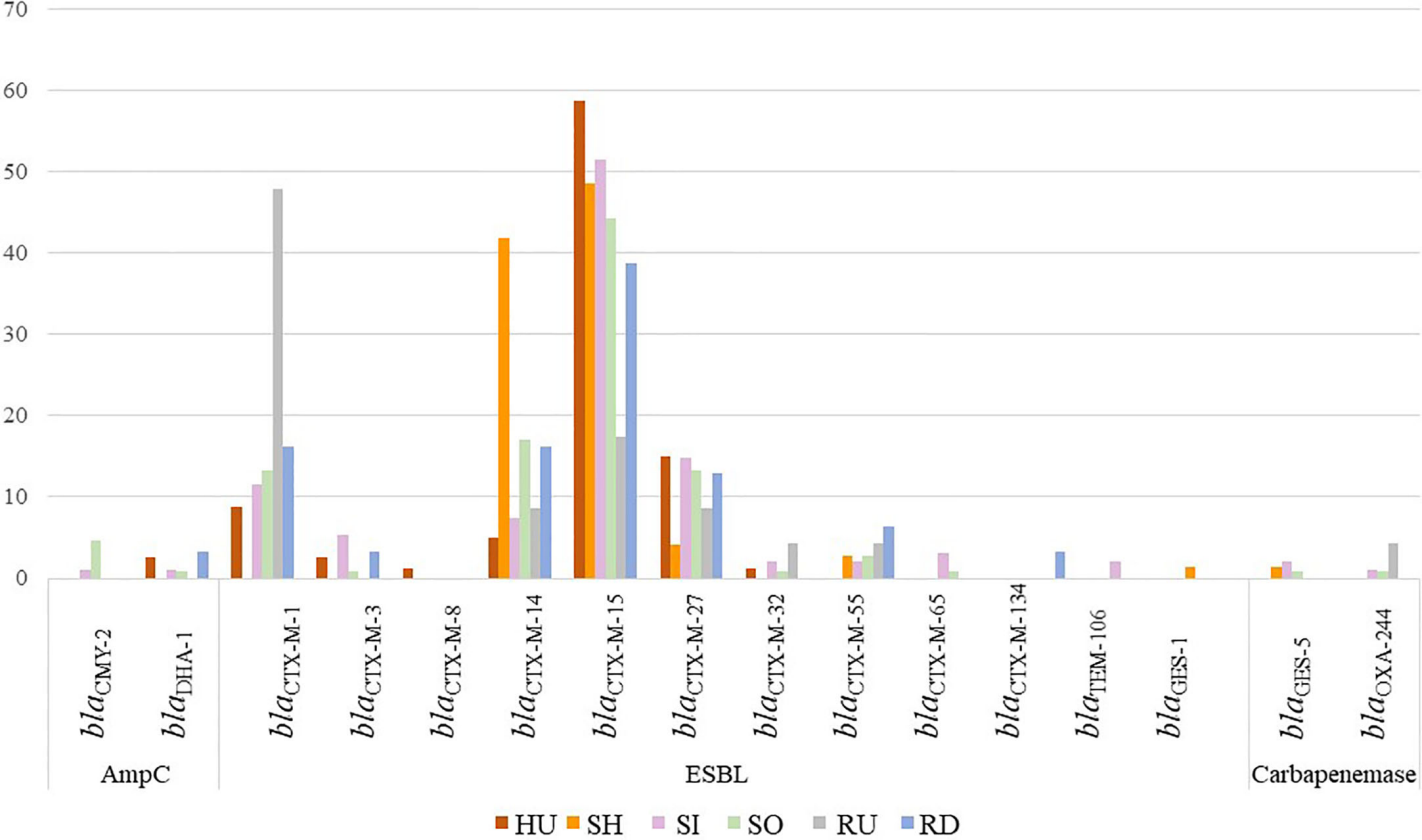
The percentage of *E. coli* isolates (n=408) with beta-lactamase encoding genes based on the source. Bar chart presents the prevalence of ESBL, AmpC, and carbapenemase-encoding genes. Source HU, UTI isolates; RD, downstream river water; RU, upstream river water; SH, hospital wastewater; SI, WWTP inflow; SO, WWTP outflow.

Obtained strains harbored (combination of) ESBL (389/408, 95%), AmpC (11/408, 3%), and carbapenemase (7/408, 2%) encoding genes (**Figure 2**). Seven strains (2%) carried narrow spectrum beta-lactamase gene *bla*_TEM-1_ (3/408) or did not contain any beta-lactamase (4/408). Nine strains carried two or more ESBL, AmpC, or carbapenemase encoding genes at once. ESBL production was mostly encoded by *bla*_CTX-M-15_ (195/408, 49%), followed by *bla*_CTX-M-14_ (68/408, 17%), *bla*_CTX-M-27_ (49/408, 12%), and *bla*_CTX-M-1_ (48/408, 12%). Genes were randomly distributed in isolates from all sources except *bla*_CTX-M-14_ which was associated mostly with wastewater. AmpC beta-lactamase producers were detected across all sources and represented by *bla*_CMY-2_ (6/408, 1%) and *bla*_DHA-1_ (5/408, 1%) genes. Carbapenemase-encoding *E. coli* included four isolates with *bla*_GES-5_ from hospital wastewater and inflow and outflow from municipal WWTPs in location A; three isolates harboring *bla*_OXA-244_ from outflow from municipal WWTPs and upstream river water in location A; and inflow to municipal WWTPs in location B. Resistance to carbapenems (mostly ertapenem) was observed among all isolates harboring *bla*_GES-5_, one out of three isolate with *bla*_OXA-244_ and, after MIC testing, also in 11 isolates without detection of any corresponding gene (**Supplement Table 2**).

A total of 73 variants of ARGs encoding resistance to 11 different antibiotic groups with a median of six ARGs per isolate were found. Clinical and upstream river water isolates showed the highest rate of ARGs (average seven genes/isolate) while those from downstream river water had the lowest number of ARGs (average four genes/isolate). ARGs were associated with resistance to sulfonamides (62%), aminoglycosides (61%), trimethoprim (53%), tetracyclines (43%), group of macrolides and lincosamides (35%), fluoroquinolones (32%), phenicols (21%), and others (<4%) (**Supplement Table 2**). At least one chromosomal point mutation known to be responsible for resistance to quinolones was found in 72% (n=292) of isolates. Particular combinations of point mutations were driven mostly by ST (**Supplement Table 2**). Mutations with unknown effect in *ampC* promoter p.P6L (resistance to beta-lactams), *pmrA* and *pmrB* genes (colistin resistance), and in *parC* and *gyrA* genes (quinolones) were observed (list of point mutations in **Supplement Table 2**).

A discrepancy between phenotype and genotypic resistances was detected in 43% of isolates. Except for colistin, discrepancies were related to all tested antibiotics. The highest discordance (more than 7% of isolates) was observed for ciprofloxacin (58/408; 14%), chloramphenicol (31/408; 8%), ceftazidime (31/408; 8%), netilmicin (29/408; 7%), and cefepime (30/408; 7%). The most common issue was the presence of genetic markers (GM) which were supposed to provide resistance to respective antibiotics without observing the resistance on the phenotypic level. For ciprofloxacin, 57 isolates showed susceptibility in thr presence of GM and one isolate was resistant in the absence of GM. Only 38 out of 57 cases of discordance for susceptible isolates with GM were related to the presence of single GM while the other 19 isolates carried at least two GM. The GM in discordant isolates were mainly represented by various mutations in *gyrA* (n=36), mostly *gyrA* p.S83L (n=27), but also the presence of *qnrS1* (n=19) or *qnrB4* (n=3) genes. Interestingly, we did not detect phenotypic resistance even for two isolates who carried six and four GM, respectively. However, as a general trend we observed that a higher number of GM is linked with higher MIC for ciprofloxacin. The isolates with MIC <0.06 mg/L carried 0.15 GM on average, those with MIC >8 mg/L carried 4.52 GM. Isolates with middle values including those within susceptible or intermediate (0.13-0.5 mg/L) and resistant (1-4 mg/L) had 0.88-1.36 and 1.44-3 GM on average, respectively. Similarly, for aminoglycoside antibiotic netilmicin, we detected 29 cases of GM without phenotypic resistance mainly related to genes *aph(3’’)-Ib* (n=12), *aac(3)-IId* (n=15), and *aph(6)-Id* (n=11) while three isolates carried a combination of five genes encoding resistance to aminoglycosides (not all of them to netilmicin specifically). On the other hand, for chloramphenicol, a similar proportion of isolates with resistance but no relevant GM (14 cases) was shown as the opposite case where GM is present but resistance is not observed (18 cases). The latter was related predominantly to *cmlA1* (11) and *catA1* (5) genes. Our results showed that even genes from *bla*_CTX-M_ family which are globally carefully monitored as the most common mechanism of resistance for extended spectrum beta-lactams did not always have resistance to ceftazidime and cefepime as was expected. These include *bla*_CTX-M-14_ (16 cases out of 68 isolates which carried this gene), *bla*_CTX-M-15_ (13/197), *bla*_CTX-M-1_ (5/48), *bla*_CTX-M-27_ (6/27), and also one case for *bla*_CTX-M-3_ and *bla*_CTX-M-55_ which were overall detected in ten or fewer isolates.

### *E. coli* population structure and pathogenic strains

Strains were assigned into nine different phylogenetic groups (PG), namely A (109/408, 27%), A/B1 (4/408, 1%), B1 (49/408, 12%), B2 (112/408, 27%), C (2/408, 1%), D (116/408, 28%), E (4/408, 1%), F (8/408, 2%), and G (4/408, 1%) (**Supplement Table 2**). A total of 74 different STs were found among isolates. The most prevalent was ST131 (n=89), which predominated in clinical isolates (39/80; 49%). In hospital wastewater ST38 (n=50), ST1972 (n=26), and ST10 (n=20) predominated.

In total, 165 (40%) *E. coli* isolates belonged to typical ExPEC-associated ST with a predominance of ST38 (12/61, 20%), ST69 (22/38, 58%), ST127 (5/5, 100%), ST131 (79/80, 99%), ST1193 (10/10, 100%), and ST4580 (6/6, 100%). The occurrence of ExPEC was highest among clinical isolates (75%) and upstream river water (57%) and the least among hospital wastewater (1%) and downstream river water (19%) strains (**Supplement Table 2**).

A statistically significant difference between total number of selected VAGs per isolate was detected only between clinical strains and hospital wastewaters, and hospital wastewaters and inflow to municipal WWTPs (p=<0.05) (**Supplement Table 2**).

### Plasmids associated with *E. coli* isolates

Altogether 54 different plasmid replicons were found in the sequenced isolates. At least one plasmid replicon was detected in 92% (n=378/408) of isolates with a median of three replicons per isolate. Plasmid replicon IncFIB (216/408, 53%) predominated in all sample sources except for hospital wastewaters where IncHI2 (40/73, 55%) plasmids prevailed. The most common F-type plasmids were F2:A-:B- (43/408, 10%), F29:A-:B10 (26/408, 6%), F1:A2:B20 (16/408, 4%), and F1:A1:B16 (13/408, 3%). Isolates with certain STs, serotypes, locations, and sources often showed the same RST.

**Figure 3:**
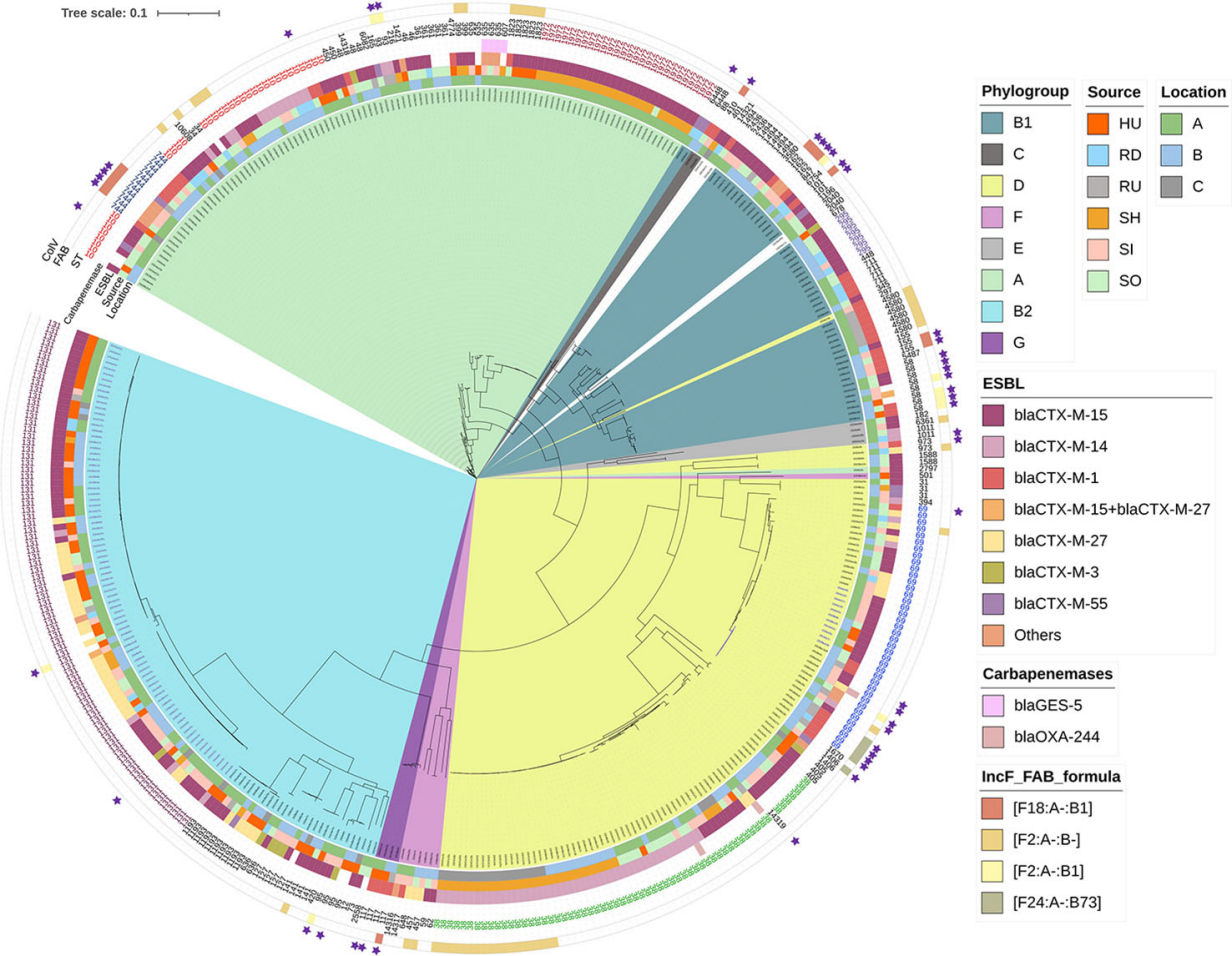
Phylogenetic tree of *E. coli* isolates based on SNP analysis. See legend for phylogenetic group, location, source, variant of CTX-M beta-lactamase (Others covers: *bla*_TEM-106_, *bla*_CTX-M-8_, *bla*_CTX-M-65_, *bla*_CTX-M-32_, *bla*_CTX-M-134_), presence of genes encoding resistance to carbapenems, STs, four most prevalent STs of F type plasmids (FAB formulas), and presence of ColV plasmids (purple stars). Colored STs were repeatedly mentioned in the manuscript. Read coding as: sampling period (1, 2), HU, UTI isolates; RD, downstream river water; RU, upstream river water; SH, hospital wastewater; SI, WWTP inflow; SO - WWTP outflow, location (A–C), number of isolates, cultivation on plate with antibiotics (c – cefotaxime/m – meropenem), variant of isolate (optionally, if from one plate two morphologically different colonies were taken).

ColV plasmids were present in 45/408 (11%) of isolates and were randomly distributed over all sources with the highest prevalence among upstream river water (30%) and inflow to municipal WWTPs (18%) and least between clinical isolates (9%). No ColV plasmids were detected among hospital wastewater isolates. ColV plasmids belonged to specific plasmid ST (**Figure 3**) with predominance of F18:A-:B1 (n=11), F2:A-:B1 (n=10), and F24:A-:B73 (n=5). They were distributed across *E. coli* genotypes with no apparent links between the particular plasmid type and *E. coli* ST. ColV plasmids were predominantly found in ST58 (7/8; 88%), ST69 (9/38; 24%), ST162 (3/3; 100%), and ST744 (4/11; 36%) (**Supplement Table 2**).

### Phylogenetic relationship of resistant *E. coli* isolates

Phylogenetic analysis confirmed the overall low genomic similarity between isolates in our collection (SNP range 2–132,281, **Figure 3**) with the exception of a few cases where significant similarities were found. Highly related isolates coming from different sources were found in the case of six ST744 isolates from municipal WWTP inflow and outflow in two locations with 3-9 SNP difference. Three isolates of ST10 from municipal WWTPs outflow and downstream river water in location B differed in 3-5 SNP only. Seven isolates of ST295 with SNP ranging from 0 to 18 from municipal WWTP inflow and outflow as well as from downstream river water were detected at location B (**Supplement Table 3**).

Individual phylogenetic trees for lineages predominating in our collection (ST10, ST38, ST69, ST131, and ST1972) were constructed and SNP matrices were computed (**Supplement Table 3**).

#### ST131

(n=80; SNP range 1 – 16,369; average 7,567 SNP; **Supplement Image 1**) strains were assigned as ExPEC (n=79/80, 99%) and divided into clades A, B, and C. Strains were assigned into virotypes A (n=9), C1 (n=4), C2 (n=3), C3 (n=1), E (n=21), F (1), and non-typable (n=11, 15%). Isolates within clade A (violet; n=28/80; SNP range 3-2,497; average 671) were detected in all sources but most of them came from wastewater (n=19/24). They contained the *fimH41* (n=23/28) or *fimH89* (n=5/28) allele, O16:H5 serotype, and harbored ESBL-encoding genes *bla*_CTX-M-27_ (n=13/27) or *bla*_CTX-M-15_ (n=11/27), and F29:A-:B10 (n=21/27) or F1:A1:B1 (n=4/27) plasmids. Clade B (black) was represented by a single isolate which carried ColV plasmid but not ESBL encoding genes. Clade C (green; n=51/80; SNP range 1 – 2,651; average 838 SNP) had the *fimH30* allele, O25:H4 serotype (n=50/51), and was divided into three sub-groups. C0 formed a single strain harboring both *bla*_CTX-M-15_ and *bla*_CTX-M-27_ and F29:A-:B10 plasmid replicon. C1 (n=16, 6 – 471 SNP) harbored mostly *bla*_CTX-M-27_ gene (n=16) and F1:A2:B20 (n=14/16) plasmids while C2 (n=35; 1 – 16,294 SNP) was mostly associated with *bla*_CTX-M-15_ (n=33) and FIA replicons with no particular family.

#### ST10

(n=33; SNP in the range 0 – 25,832; average 9230; **Supplement Image 2**) was detected in all sampled sources and locations. Strains were divided into six clusters where only cluster 6b contained phylogenetically related isolates (violet; n=16; 0-134 SNP; average 90); strains had *fimH24* (n=14/16), O9:H9, and contained *bla*_CTX-M-14_ (11/16) gene and F1:A1:B16 (12/16) replicons.

#### ST38

(n=61; 2 – 16,293 SNP; average 5648, **Supplement Image 3**) was found in all sources and represented a heterogenous group. Strains were mostly of *fimH5* type and carried *bla*_CTX-M-14_ (n=44/61, 72%) except clusters 3c and 3d (orange) where *bla*_CTX-M-15_ (n=15/61, 25%) and *fimH54* predominated. Strains in cluster 1 (n=31; SNP range 2 – 240, average 92) were mostly from hospital wastewater; cluster 1a (violet) isolates were from location C (n=31; SNP range 6 – 201; average 23) and harbored no ARGs apart from *bla*_CTX-M-15_ and F2:A-:B plasmid (n=18/31, 58%), whereas strains in cluster 1b (red) were from locations A and B (SNP range 2 – 240; average 90) and frequently contained four ARGs (including mobile colistin resistance mediated by *mcr-9*) and HI2 replicon. Strains in cluster 2 were often found in WWTPs and assigned as ExPECs (n=12/61, 20%). Strains were more diverse in ARGs and plasmid replicons than in cluster 1a. Two isolates from location A (clusters 2, 3b) carried *bla*_OXA-244_.

#### ST69

(n=38; 1,444 sites; SNP in the range 7 – 12,159, average 6416, **Supplement Image 4**) were divided into three main clusters. Strains originated mostly from wastewater and river water, except strains from cluster 1 (violet; n=11/38, SNP range 7 – 77; average 32) which were also detected among clinical isolates. No particular RST or ESBL gene were connected with ST69, however isolates from cluster 1 contained *bla*_CTX-M-15_ (n=11/11, 100%). Moreover, one isolate (1SIBe14mer) within cluster 2b carried *bla*_OXA-244_ which was not manifested phenotypically. The presence of ExPECs (n=22/38, 58%) was randomly distributed but mostly connected to clusters 1 and 3. Presence of ColV plasmids (n=9/38, 23%) was associated mainly with cluster 2b.

#### ST1972

(n=31; SNP range 32 – 1,314; average 496 SNP, **Supplement Image 5**) within this study were obtained in location A (n=26) and were compared to strains obtained in our previous study (n=5) in 2016. The strains were divided into three clusters and the minimal SNP difference between those from 2016 and this study was 230 SNP. Strains in clusters 1, 2, 3a, and 3b (violet) were collected from hospital wastewater and municipal WWTP outflow within this study and shared similar characteristics. They predominantly harbored *bla*_CTX-M-15_ (n=26/26, 100%) and *bla*_OXA-10_ (n=21/26, 80%) and had a median of eight ARGs replicons per isolate. Cluster 3c formed isolates from a previous study forced on carbapenemase producers (green; n=5; SNP range 106 – 381; average 192 SNP, **Supplement Image 4**). They harbored *bla*_GES-5_ (n=5/5, 100%) and additionally only *qnrS2* gene. ST1972 had serotype O-:H12, fimbriae type *fimH305*, and contained H2 (27/31, 87%) and X1 (n=18/31, 58%) replicons. In contrast to the other clusters, all isolates from 3c missed FIB_K_ replicon.

## Discussion

The aim of this study was to explore the spread of antibiotic-resistant *E. coli* isolates from UTI, hospital wastewater, municipal WWTPs, and adjacent rivers using deep genomic and phenotypic characterization. We showed that clinical, wastewater, and river water isolates harbored diverse STs with variable antibiotic resistance profiles and genetic background but UTI strains formed a minor proportion of the resistant bacteria in hospital sewage. Dissemination of hospital-associated strains through municipal WWTPs was not confirmed within this study but pathogenic lineages were also present in river water.

Most isolates obtained from all sources showed MDR profile and produced ESBL but, in general, UTI isolates were resistant to more antibiotics than strains obtained from wastewater and river water samples. Almost half of the isolates showed some level of discordance between the phenotypic resistance and WGS-based antibiotic resistance prediction for most antibiotics. Discordance was observed in the case of ESBL genes and phenotypic resistance to beta-lactams cefepime and ceftazidime. The CTX-M enzymes confer high-level resistance to cefotaxime, ceftriaxone, and aztreonam, but have only marginal effects on MICs of ceftazidime, depending on the gene variant (Tzouvelekis et al., 2000). The most problematic were CTX-M enzymes from group 9 (*bla*_CTX-M-14_ and *bla*_CTX-M-27_) where only 77% of isolates carrying the gene showed resistance to ceftazidime. A similar observation was made by Williamson et al., 2012 and Wyrsch et al. (2022) where only 7% and 14% of isolates carrying *bla*_CTX-M-14,_ respectively, were resistant to ceftazidime. This discordance can be explained by a generally lower level of hydrolytic activity of CTX-M-14 against ceftazidime (Bonnet et al., 2003). Globally, the most prevalent ESBL-encoding gene from CTX-M group 1, *bla*_CTX-M-15_ (Bevan et al., 2017), confers resistance to both cefotaxime and ceftazidime (Poirel et al., 2002) but in our study some level of discordance (7%) was observed.

The discordance between phenotypic results and detected genetic markers for ciprofloxacin which was most commonly observed in our study is a known phenomenon described previously (Rebelo et al., 2022). While these GM have been proven to increase MIC for ciprofloxacin, they do not always reach the breakpoint values for resistance so the system of binary classification is not optimal here as pointed out previously (Rebelo et al., 2022). Interestingly, we commonly detected mutations of *gyrA*, such as *gyrA* p.S83L, in susceptible isolates while these types of mutations alone are classified to provide the base for ciprofloxacin resistance in *E. Coli* according to ResFinder. In general, we observed that higher MIC is linked with a higher average of GM which corresponds with previous studies (Vila et al., 1994; Rebelo et al., 2022). On the other hand, very close values were observed for isolates still classified as susceptible with MIC 0.25 mg/L (with an average 1.39 GM) and intermediate phenotype 0.5 mg/L (with an average 1.36 GM) which highlights that the quantity of GM alone is also not an ideal predictor as these genetic markers are known to not have the same impact so their influence, especially in combinations, should be further studied. These data are crucial as they are the basic requirement for developing a new type of fluoroquinolones resistance prediction scheme which is critically needed.

Seven carbapenemase-producing isolates were obtained from all sources except for UTI and river downstream WWTP. Several isolates of these carbapenemase producers belonged to the same ST but they were genetically distinct based on the number of SNP, suggesting their origin from multiple sources. Surprisingly, only two generally rarely reported carbapenemases in *Enterobacterales*, GES-5 and OXA-244, were detected and their phenotypic manifestation to carbapenems differed according to the corresponding carbapenamase gene. GES-5 is usually associated only with reduced susceptibility to carbapenems rather than full resistance but shows good carbapenem-hydrolyzing activity (Cantón et al., 2012) and it was manifested phenotypically (in case of ertapenem only) in all four isolates in our study. On the other hand, OXA-244 is a derivative of OXA-48 exhibiting low carbapenem MIC values and weak carbapenemase activity, thus making their detection quite challenging in clinical laboratories (Masseron et al., 2020). All isolates with OXA-244 from our study were susceptible to meropenem and only one of them showed resistance to ertapenem. To select for carbapenemase producers, we used in-house media with a low amount of meropenem as our previous observation suggested low concentrations of carbapenemase-producing bacteria in environmental samples (Dolejska et al., 2016). Commercially available media with higher concentrations of carbapenems may result in missing some variants of carbapenemase genes with lower carbapenemase activity as previously observed (Dolejska et al., 2016). Additionally, as was reported (Fawaz et al., 2019), meropenem is unstable in solution and when the concentration in the media drops, the selective effect rapidly decreases. This selection method gave us a high number of isolates with only reduced susceptibility to carbapenems rather than resistance, therefore, we decided to apply PCR to select for isolate carrying carbapenemase-encoding genes prior to WGS. This two-step approach proved to be efficient as only 3% of isolates growing on media with meropenem contained carbapenemase-encoding genes.

One of the main goals of this study was to reveal the link between the community structure of UTI, hospital wastewater, municipal WWTPs, and river water. As Buelow et al. (2018) previously reported, we observed only a minor impact of UTI strains to the composition of hospital wastewater. Distribution of *E. coli* genotypes revealed the predominance of ST131 in most sources, especially in UTI strains where 47% of the isolates belonged to ST131. Several studies reported increased virulence and reduced antibiotic resistance among UTI- causing *E. coli* (Horcajada et al., 2005; Johnson et al., 2002) Regarding the bias of this study in which only cephalosporin- and carbapenem-resistant isolates were selected by the use of antibiotic-supplemented media, we did not observe any difference between antibiotic resistance or VAGs among ST131 isolates. Even strains within traditionally antibiotic-sensitive ST131 clade A (Pitout & Finn, 2020) showed similar patterns in ARGs and VAGs as was observed in clade C strains. Other predominant lineages observed in our study, such as ST10, ST38, or ST69, were not predominantly detected in UTI but more often in wastewater. As was reported, these strains could also successfully colonize gut system and/or cause diarrhea (Matsui et al., 2020; Zhou et al., 2020) and therefore could have been associated with feces instead of UTI. ST10 and ST69 have been documented as the dominant *E. coli* population in fecal samples of healthy people (Matsui et al., 2020), therefore its higher presence in municipal WWTPs compared to hospital sewage suggests their community origin.

Despite the overall high genetic diversity between the strains characterized in this study, some of our data suggest that *E. coli* of certain lineages are transmitted from hospitals *via* WWTPs to rivers. We detected a quite rare ST1972 (only 19 other strains in Enterobase to date 6.3.2023; Zhou et al., 2020) in both hospital wastewater and municipal WWTPs outflow. Moreover, we compared our results with our recent study and found out that this lineage was present in wastewaters in the very same location even in 2016. Although the clonal spread was not confirmed as the isolates differed in 230 SNP on average, it suggests that ST1972, frequently found in raw hospital sewage in location A (87% isolates belonged to this ST), is likely a hospital-adapted ST spreading *via* wastewater. Phylogenetic analysis of ST10 and ST295 strains from WWTPs and rivers downstream from location B provided the evidence of transmission of resistant strains *via* wastewater to the environment as the isolates differed by less than 10 SNP. Similarly, highly related isolates of ST744 from inflow and outflow of WWTP proved that it can resist the wastewater treatment process.

Regardless of our expectations, we found only a limited number of ExPEC strains in hospital wastewater and a surprisingly high number of ExPECs in upstream river water. Firstly, the main source of hospital wastewater strains was probably not UTI (e.g. ST131) as we demonstrated in this study, but rather stool samples (Matsui et al., 2020). Secondly, evaluation of strains as ExPECs is problematic and not clearly defined by a set of VAGs which could cover all strains (Zhi et al., 2020) Thirdly, data obtained from upstream river water were distorted because only isolates from the first sampling period right after the flood were obtained. Therefore, the frequent occurrence of ExPECs in river samples could be caused by arising bacteria present in the sediment, as was reported previously (Hanna et al., 2020), or releasing untreated wastewater from an overloaded sewage network (Abraham, 2011).

The spectrum of plasmid replicon in the sequenced isolates was quite diverse but F type plasmids predominated. We investigated the possible links between the plasmid type defined by RST, *E. coli* ST, and the source. Generally, various RSTs were present across STs with some minor associations of F type plasmid and *E. coli* genotype. Predominant F2:A-:B-, which was previously found in animals (Chen et al., 2014) and clinical isolates (Ho et al., 2013), was in our dataset mostly associated with ST10, ST38, and ST69 of hospital and municipal wastewaters. Plasmid replicon F1:A1:B16 was previously found in ST131 (Paulshus et al., 2019; Tóth et al., 2022) while ST10 was its main carrier within this study. On the other hand, pUTI89-like plasmid F29:A−:B10, linked to ExPECs and uropathogenic *E. coli.* (Li et al., 2021), was almost exclusively connected to ST131 clade A as Pitout and Finn (2020) previously observed. The only two exceptions of other STs carrying pUTI-89 formed ST95 from WWTP inflow, in which this plasmid type was previously reported (Cummins et al., 2022), and ST405 UTI isolate. Another type of plasmid we explored was ColV plasmids, of which type plasmids are associated especially with avian pathogens and represent a link between poultry and human health risks (Skyberg et al., 2006; Reid et al., 2022) ColV plasmids were documented to be present in ExPECs (Reid et al., 2022) and especially connected to UTI, but in our study we found only 1% of ExPECs and 9% of UTI isolates to carry these plasmids. Similarly, as Reid et al. (2022) reported, ColV plasmids were tightly associated with specific STs or RSTs. Interestingly, we did not observe ST with specific RST harboring ColV in our study, except for F24:A-:B73 where the majority (4/6) of ColV-harboring strains were formed by ST69.

In this study we encountered limitations which could influence the final results. Firstly, floods that occurred before the first sampling period could cause the increased number of detected *E. coli* isolates in river water during the first sampling. As was documented by Lee et al. (2022), untreated combined sewage distributed from WWTPs contributes to increased levels of ARB in rivers and elevated resistance risk factors persist for up to 22 h. Secondly, we decided to use grab samples of both hospital and municipal wastewater and river water fully aware that it provides only a snapshot of the population in time. This could lead to an underestimation of the impact of UTI-associated bacteria in hospital wastewater. Composite samples generated by automatic samplers would cover a larger time period, as would setting sampling time intervals, but we wanted to prevent the possibility of the overgrowth of bacteria in our experiments. Thirdly, in location C we collected only isolates from UTI and hospital sewage but not from WWTPs input and output and river water.

## Conclusion

In summary, this study highlights the influence of urban wastewaters on the receiving environment in the context of dissemination of ARBs and ARGs. Being aware of bias driven by selective cultivation on antibiotic-supplemented media, all tested *E. coli* strains (up to 30 from each sample) exhibited a broad range of antibiotic resistances regardless of sampling period, location, or sources. SNP analysis provided evidence of transmission of several lineages from WWTPs to downstream river water. Moreover, transfer of strains from hospital wastewaters *via* WWTPs and the presence of specific, phylogenetically related ST1972 with nosocomial origin 5 months apart in wastewater indicates that strong measures should be implemented to prevent the worsening of an already alarming situation in antibiotic resistance dissemination.

## Data availability statement

The datasets presented in this study can be found in online repositories. The names of the repository/repositories and accession number(s) can be found below:.https://www.ncbi.nlm.nih.gov/genbank/, PRJNA938932.

## Author contributions

LD-G, IS, and MD conducted the research and designed the experiments. LD-G, JL, IS, LN, KV, KC, and MK performed the experiment. LD-G, JL, IS, KN, JK, and JP provided the data analysis. LD-G wrote the manuscript. MD and others provided revisions. All authors contributed to the article and approved the submitted version.
