# Supplementary figures and images for: Hospital and community wastewater as a source of multidrug-resistant ESBL-producing Escherichia coli
Source: Front Cell Infect Microbiol. 2023 May 15;13:1184081. doi: 10.3389/fcimb.2023.1184081 (PMC10225658; doi:10.3389/fcimb.2023.1184081)

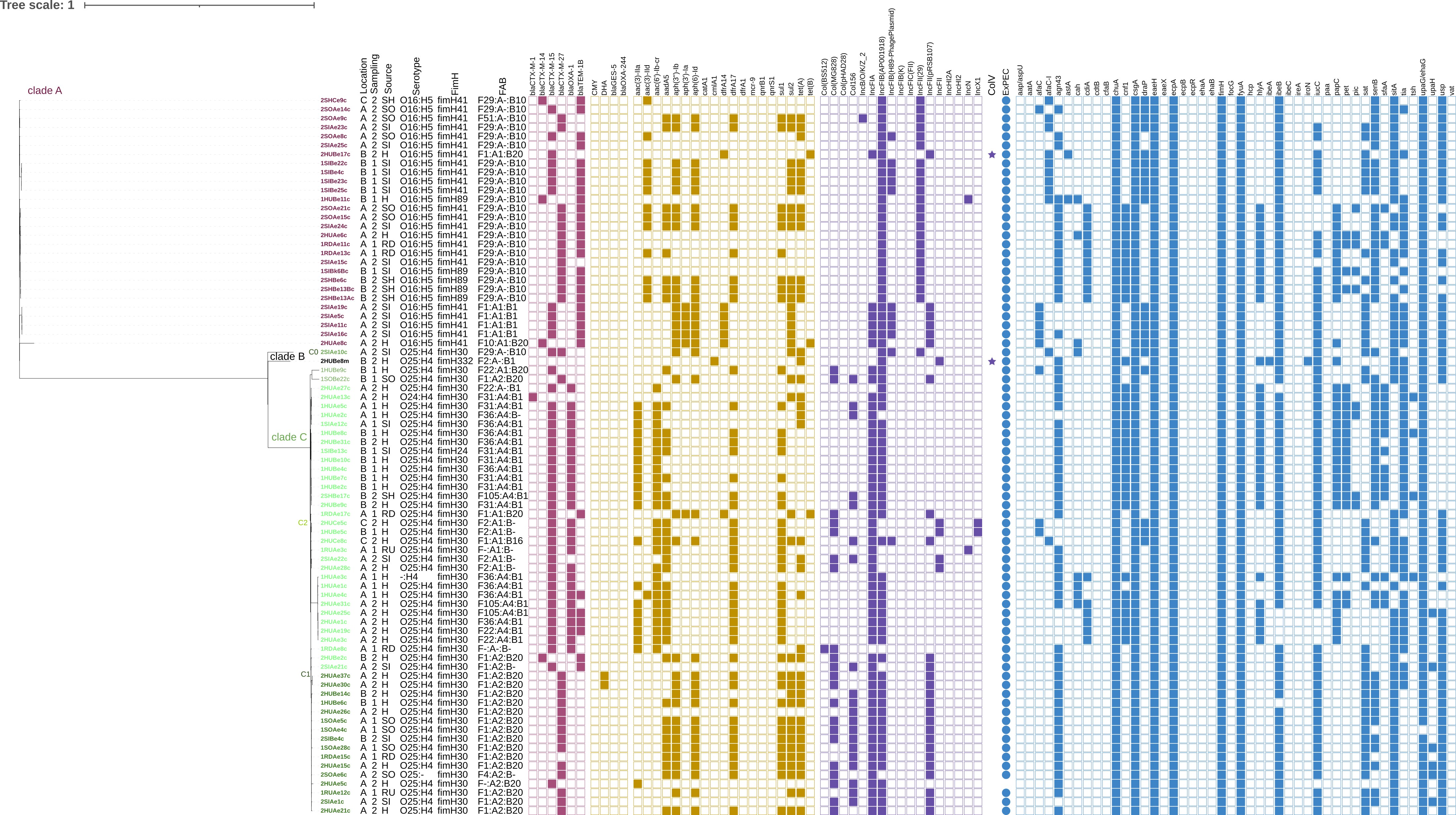

Supplement: Supplementary file 1 [file Image_1.jpeg]

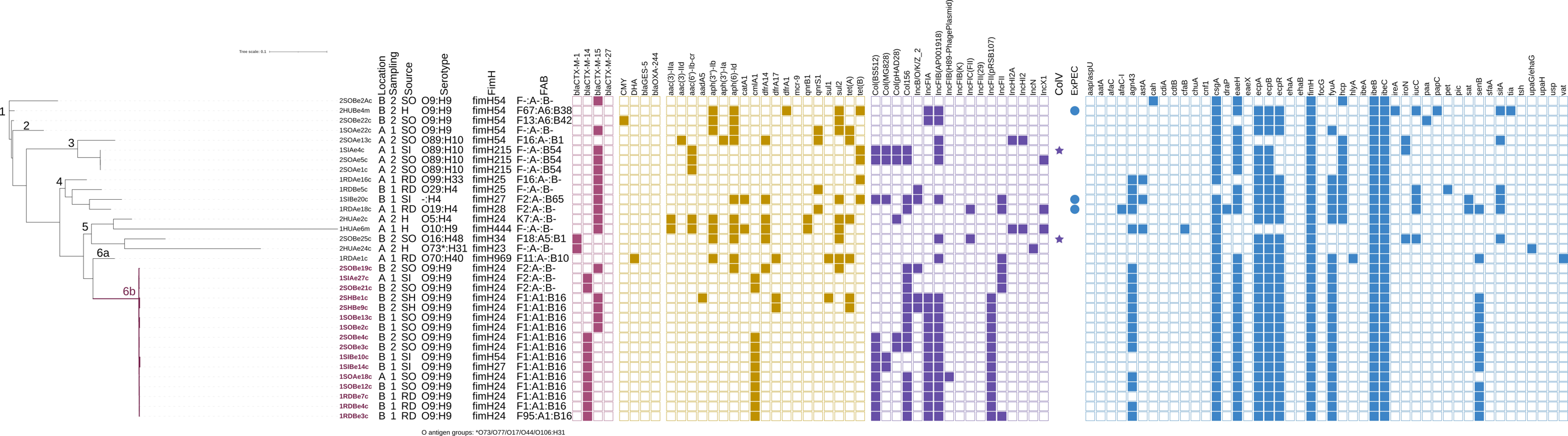

Supplement: Supplementary file 2 [file Image_2.jpeg]

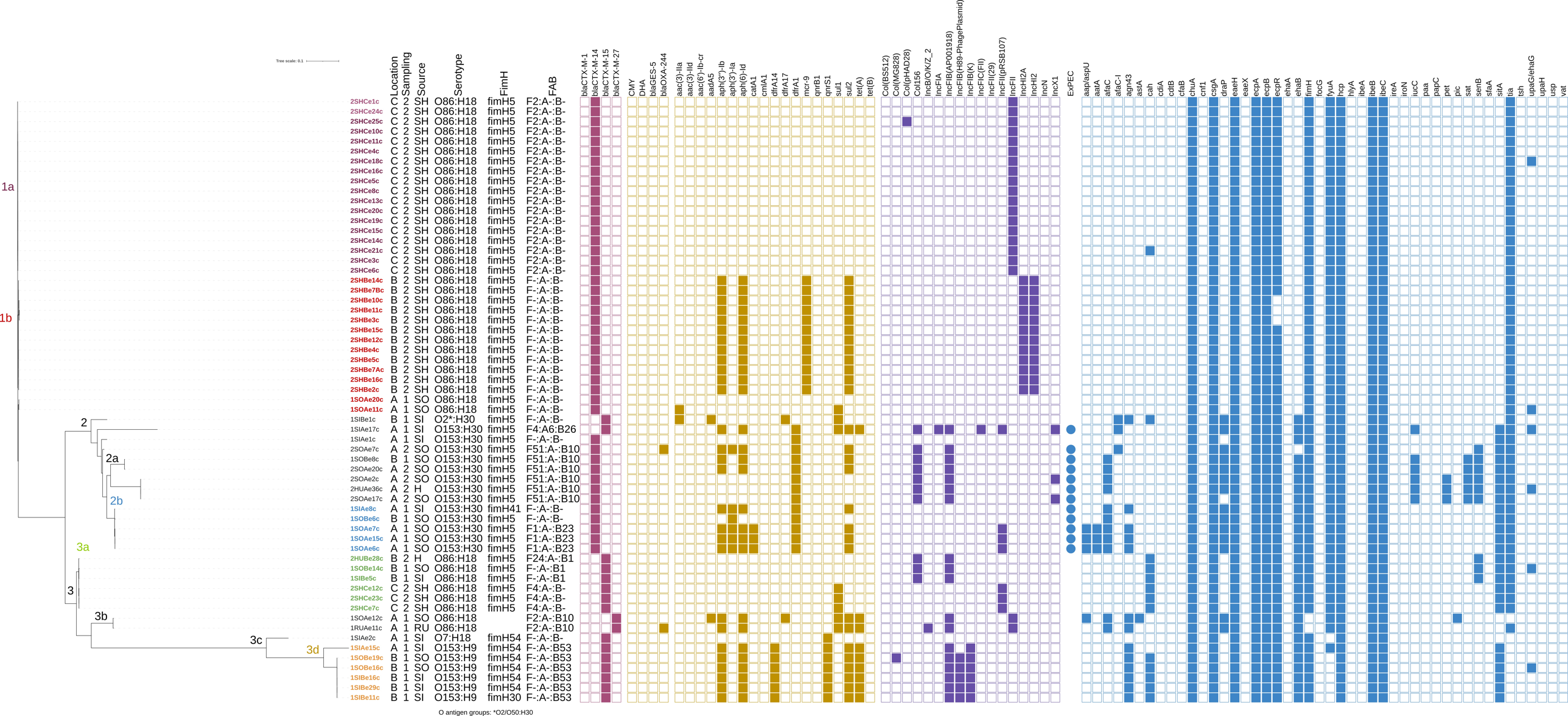

Supplement: Supplementary file 3 [file Image_3.jpeg]

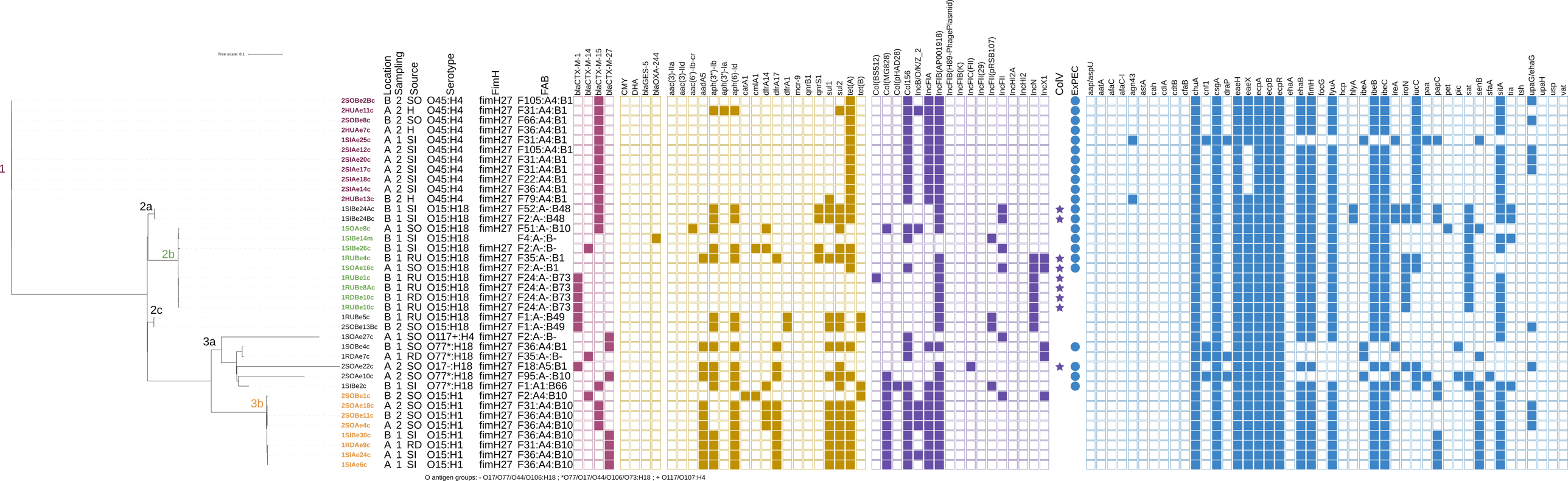

Supplement: Supplementary file 4 [file Image_4.jpeg]

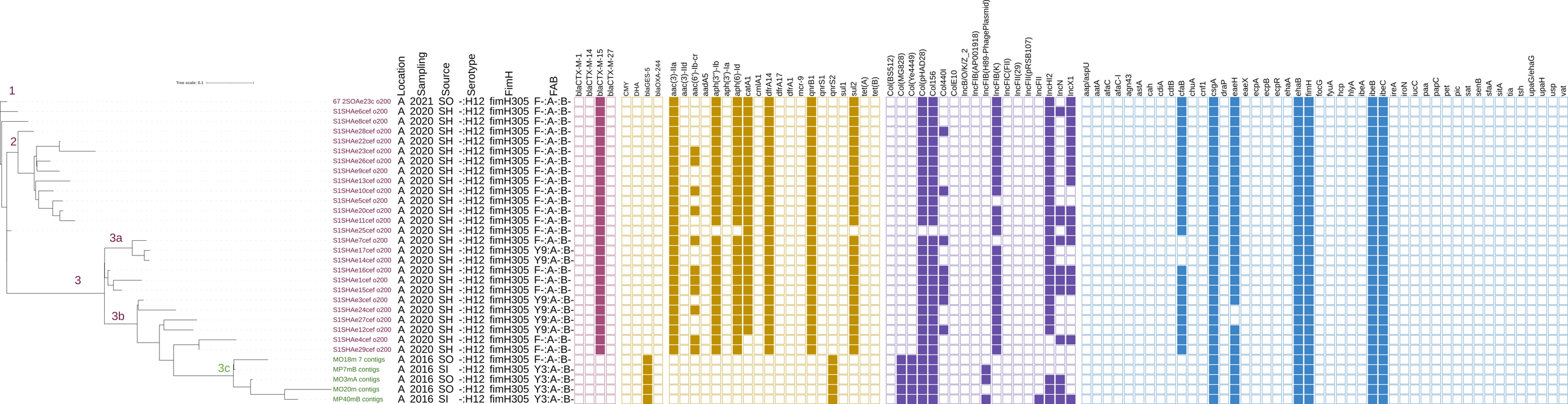

Supplement: Supplementary file 5 [file Image_5.jpeg]
